# Norovirus Binding to Ligands Beyond Histo-Blood Group Antigens

**DOI:** 10.3389/fmicb.2017.02549

**Published:** 2017-12-21

**Authors:** Erin A. Almand, Matthew D. Moore, Lee-Ann Jaykus

**Affiliations:** ^1^Department of Food, Bioprocessing and Nutrition Sciences, North Carolina State University, Raleigh, NC, United States; ^2^Department of Food Science, University of Massachusetts, Amherst, MA, United States

**Keywords:** norovirus, histo-blood group antigens, virus–bacteria interaction, transkingdom, enteric virus

## Abstract

Histo-blood group antigens (HBGAs) are commonly accepted as the cellular receptors for human norovirus. However, some human noroviruses have been found not to bind any HBGA ligand, suggesting potential additional co-factors. Some ligands have been found to bind noroviruses and have the potential to be additional cellular receptors/attachment factors for human norovirus or inhibitors of the HBGA interaction. The studies identifying these mostly characterize different chemical, human, food, or bacterial components and their effect on norovirus binding and infection, although the mechanism of interaction is unknown in many cases. This review seeks to supplement the already well-covered HBGA-norovirus literature by covering non-HBGA human norovirus ligands and inhibitors to provide investigators with a more comprehensive view of norovirus ligands.

## Introduction

Human noroviruses are a leading cause of viral gastroenteritis worldwide, estimated to account for over 685 million illnesses globally every year (Kirk et al., 2015). These viruses are highly diverse members of the *Caliciviridae* family, with over 30 strains divided into seven genogroups (GI and GII are the primary infectious groups in humans), and further divided into genotypes (Kroneman et al., 2013). The genotype GII.4 is the most prevalent outbreak genotype, with new pandemic strains emerging and circulating globally every few years, partially assisted by its rapid mutation rate (Debbink et al., 2013). Genotypes are further divided into strains usually based on the location of the first isolate of the strain (i.e., GII.4 Sydney), adding complexity to these extremely variable pathogens (Ramani et al., 2014).

Many aspects related to the infection cycle of human norovirus remain elusive to researchers. Despite recent advances in the field (Karst et al., 2014; Thorne and Goodfellow, 2014; Ettayebi et al., 2016), a number of questions still remain–especially with regards to the binding and uncoating process. Over 10 years ago, researchers identified putative human norovirus cellular receptors called histo-blood group antigens (HBGAs) (Harrington et al., 2002, 2004; Marionneau et al., 2002). HBGAs are complex terminal carbohydrates present on cells (i.e., red blood cells and mucosal epithelium) and secreted into bodily fluids (i.e., saliva, intestinal secretions) in many cases (Marionneau et al., 2001). These carbohydrates are generated from disaccharide precursors that then receive stepwise addition of monosaccharides with different glycosyltransferases in specific locations that determine the different types of HBGA. The HBGA system is very complex, and for the purposes of this review only a specific subset of HBGAs relevant to human norovirus binding will be discussed; specifically the ABH and Lewis systems. Interested readers are referred to Marionneau et al. (2001) and de Graaf et al. (2016) for more comprehensive reviews of HBGAs. Specifically, the enzymes to be discussed are fucosyltransferases, encoded by the *FUT1* and *FUT2* genes that add a fucose in an α1,2 linkage to the terminal galactose of the disaccharide precursor (types 1–4) at different locations. The product is called the H antigen and can be further extended by the A and B enzymes that add *N*-acetylgalactosamine and galactose, respectively, in an α1,3 linkage to generate A or B HBGA types. Lewis antigens involve additional fucosyltransferases that add fucose in α1,3 or α1,4 linkages to *N*-acetylglucosamine on the precursor to generate the different Lewis antigens. For instance, Lewis a and x are generated when a fucose is added with α1,4 and α1,3 linkages to the *N*-acetylglucosamine of the disaccharide precursor, respectively, while Lewis b and y are generated if that same fucose addition is added to an H antigen. Individuals with two non-functional *FUT2* alleles do not have HBGAs in their saliva and on certain epithelial cells, and are considered “non-secretors” (Marionneau et al., 2001).

The association of HBGAs with norovirus was partially borne of epidemiological studies suggesting patients with type O blood were more susceptible to GI.1 Norwalk virus (the prototype strain) infection (Hutson et al., 2002) and empirical observations from challenge studies, as “non-secretors” remained resistant to infection upon challenge with this virus (Lindesmith et al., 2003). Selective Norwalk capsid binding to intestinal epithelial cells of individuals with this intact HBGA enzyme also supported these observations (Marionneau et al., 2002). Subsequent studies demonstrated the ability of numerous other norovirus strains to bind a variety of different HBGA types, generally in genotype- or strain-specific manners (Tan and Jiang, 2005). These original findings shortly became the foundation for a much more complex virus-receptor relationship.

Even though strong evidence has existed implicating HBGAs in human norovirus infection, additional evidence has been reported that suggests other co-factors or receptors may exist, at least for specific strains. This question has been historically relevant, as a simple *in vitro* cultivation (Duizer et al., 2004) or ideal animal model (Moore et al., 2015) had not yet been identified, despite comprehensive effort. Guix et al. (2007) presented evidence suggesting that the major roadblock in attaining such a model was in the binding and/or uncoating stage, which would involve HBGAs in part. Purified norovirus RNA was transfected into cells and resulted in viral replication through one cycle, releasing packaged particles. Up-regulating expression of HBGAs via overexpression of the *FUT2* gene involved in HBGA synthesis in the cell resulted in increased binding of viral particles, but the particles could not be internalized and continue infection (Guix et al., 2007). Other suggestions of additional elements in the binding and uncoating process have also been reported. For example, an early challenge study with GII.2 Snow Mountain Virus that failed to find a correlation between infection and host ABO blood group, secretor status, or Lewis status, suggesting that Snow Mountain virus may utilize different types of HBGAs or different classes/groups of receptors. However, it should be noted the sample size of volunteers tested was fairly low (15 volunteers) (Lindesmith et al., 2005). A retrospective report did not find a statistically significant correlation between ABO blood group and seropositivity of individuals to a GII norovirus (Rockx et al., 2005). Additional reports of other norovirus strains that do not bind any of the available synthetic HBGAs or saliva (Huang et al., 2005; Shirato et al., 2008; Donaldson et al., 2010) suggest that additional modifications of HBGAs or additional binding/co-factors may be involved in norovirus binding and uncoating. A recent binding study investigating the ability of norovirus capsids to bind different intestinal cell lines and intestinal biopsies found a lack of requirement for specific HBGAs for binding. Specifically, report found that capsid binding to Caco-2 cells may involve additional components beyond HBGAs in binding and internalization, as binding was found to be related to differentiation of the cells but not HBGA expression. Additionally, capsids of the GII.1 virus that did not bind HBGAs were found to bind and become internalized in the intestinal epithelium of ileal biopsies (Murakami et al., 2013).

Recent reports of two new human norovirus cell culture models and identification of a murine norovirus receptor represent major advances in the field; however, they also reiterate the questions raised above suggesting the existence of additional co-receptors/attachment factors. Jones et al. (2014) presented evidence suggesting that HBGA-like molecules presented on enteric bacteria are a requirement or co-factor involved in human and murine norovirus infection. In the work, Jones et al. (2014) provided evidence that the enteric bacteria may aid attachment and infection of the viral particles to B cells, potentially by providing passage for the virus through the epithelial layer. Another human norovirus cell culture model was presented by Ettayebi et al. (2016), who reported productive infection of different norovirus strains using human intestinal enteroids. In this case, no bacteria or added HBGA were needed for infection of the enteroids, but addition of bile acids was required for infection with some strains and generally enhanced replication of all the strains tested (Ettayebi et al., 2016). Interestingly, the effect of secretor status on replication of GII.4 vs. GII.3 strains was different. Both GII.4 and GII.3 were capable of infecting enteroids derived from the stem cells of secretor hosts, but only GII.3 was capable of infection in enteroids derived from secretor-negative hosts (Ettayebi et al., 2016). Another recent paper presents evidence of a receptor for murine, GV, noroviruses (Orchard et al., 2016). In this case the receptor was proteinaceous, CD300lf, and it was found to be necessary for murine norovirus binding and infection in cell lines, including a human cell line with murine CD300lf cloned into it (HeLa cells). However, an unidentified co-receptor/co-factor found in serum is required in addition to CD300lf for efficient binding. Evidence suggested that it was <5 kDa, present in delipidated serum, and was resistant to boiling and proteinase K treatment. The sum of this evidence suggests that other attachment or co-factors may be involved in human norovirus pathogenesis, and while norovirus-HBGA binding has been well-reviewed (Ruvoën-Clouet et al., 2006; Donaldson et al., 2008; Tan and Jiang, 2010, 2014), we review reports of additional molecules found to specifically bind human norovirus capsids beyond HBGAs.

Other studies highlight ligands which either directly bind to human norovirus with comparable affinity as HBGAs or potentially inhibit binding, suggesting they might occupy or occlude the same pocket on the human norovirus capsid. However, it is difficult to conclude this is the case short of a crystal structure for many of these molecules as binding interactions with a number of them are multivalent, and thus confound conclusions that can be drawn from binding competition experiments. For the purposes of this review, other human norovirus ligands will be separated into a few different categories: simple chemical compounds, complex human biological components, and HBGA-like moieties. Simple chemical compounds may associate through unique binding sites or by interacting in a similar manner to HBGAs. Human components have been shown to interact with the HBGA binding pocket or unspecified regions on the capsid (Tamura et al., 2000). Many of these studies evaluated virus binding to relevant cell lines or inhibition of this binding, and in some cases the actual binding location remains unknown. The HBGA-like moieties currently important in human norovirus research may be found on food items (Tian et al., 2006; Esseili et al., 2012) and naturally occurring gut microflora (Miura et al., 2013). These moieties interact directly with the binding pocket, utilizing sugar residues similar to those found on human-derived HBGAs to bind norovirus. These categories are not necessarily mutually exclusive; for example, a number of the simple chemical components discussed are present in human cells.

## Human Norovirus Structure and HBGA Binding

The human norovirus genome is comprised of three open reading frames (ORFs). These regions code for the non-structural proteins (ORF1), major capsid protein (VP1; ORF2), and the minor capsid protein (VP2, ORF3) (Debbink et al., 2012). The VP2 protein is considered a minor capsid protein, present only in a few copies and thought to be associated in the inside of the assembled capsid (Vongpunsawad et al., 2013). The VP1 protein self-assembles into dimers and 90 dimers assemble to form the viral capsid. The protein consists of an N-terminal shell (S) domain that is responsible for maintaining the icosahedral contacts of the particle. It serves as the scaffold upon which the protruding (P) domain extends. The P domain contains an outermost P2 subdomain and a P1 subdomain that is located closer to the S domain in a 3D structure of the virus. The P2 subdomain is the most variable region of the genome, protrudes the furthest from the S domain, and is a major contributor to genetic drift and the emergence of new human norovirus outbreak strains (Donaldson et al., 2010). The particle self-assembles; thus if the VP1 (and sometimes VP2) ORF is cloned into animal cells, it can be expressed to produce a large number of assembled, non-infectious viral capsids, called virus-like particles (VLPs). VLPs have the advantage of being produced at a higher concentration *in vitro*, can be more easily purified than infectious virus from stool, and exhibit generally similar antigenic and receptor-binding properties as infectious virus; making them valuable reagents in the study of human norovirus. However, VLPs have limitations in terms of the degree of concentration and purity that is easily achievable (Koho et al., 2012). To overcome the potential hurdle of easier expression and purification, cloning the P domain can result in the formation of individual P dimers, or small collections of P dimers depending on modifications made to the N terminus of the protein (Tan and Jiang, 2012). These reagents are easy to purify as they contain a protein tag, and easier to produce at higher levels and lower expense in *Escherichia coli*. These dimers still maintain similar structure to the P dimers in the capsid, and bind HBGAs in a similar manner. These reagents are especially valuable for structural study using X-ray crystallography (Cao et al., 2007). The high mutation rate of ssRNA viruses fuels a great deal of genetic diversity (Hardy, 2005; Tan and Jiang, 2005) and allows noroviruses to alter their antigenic and receptor binding profiles in response to selective pressure. Thus, different pandemic human norovirus strains emerge every couple of years causing outbreaks worldwide (Debbink et al., 2012).

Matching these diverse viral structural motifs are a complicated group of potential carbohydrate receptors. Norovirus interacts with these traditional sugar moieties in specific binding patterns (Cao et al., 2007) categorized by the virus binding to the A/B or Lewis residues (Huang et al., 2005). Despite these broad characterizations, there are specific binding patterns by the different human norovirus strains, and some strains do not bind any HBGA as mentioned above, or if binding occurs, the methodology employed was not sensitive enough to detect it. Numerous structural reports and characterizations of different noroviruses binding to different HBGAs have been documented, and are beyond the scope of this review (Cao et al., 2007; Bu et al., 2008; Choi et al., 2008; Chen et al., 2011; Kubota et al., 2012; Singh et al., 2015). Interestingly, a recent report provides the structural basis for why some norovirus strains (two GII.1 strains and GII.2 Snow Mountain) may not strongly bind HBGAs, linking lack of binding to an aspartic acid located in the P2 subdomain, in a region that is disordered and oriented away from a fucose residue present on the HBGAs studied (Singh et al., 2016).

Generally, there are two recognized HBGA-interaction sites within the P2 subdomain of the human norovirus capsid protein, Site A and Site B. Variations within these sites lead to the major architecture of human norovirus binding; the strains that bind to ABH antigens or the strains that bind to Lewis or H antigens. Within the GII.4 genotype, strain specificity is largely determined by Site A, putatively between amino acid residues 296–298 in the GII.4 Lordsdale strain, whereas the stability and subsequent strength of the interactions is thought to be modulated by Site B located at residues 393–395 (Zakikhany et al., 2012). These regions vary across different human norovirus strains. Regardless of the location of interactions, it is believed the specificity in Site A is determined through a hydrogen-bonding array between the fucose groups or the A/B terminal sugar (*N*-acetylgalactosamine or galactose), based on HBGA preference. Site B is responsible for longer range interactions, stabilizing the molecule (Donaldson et al., 2008). Variations within these sites alter the binding specificity and strength of norovirus-HBGA interactions, and disruption of this binding pocket by a competing molecule or occlusion of the different amino acid residues reduces human norovirus binding (Tan and Jiang, 2005).

While both GI and GII genogroups bind HBGAs in the same region of the P2 domain, the actual binding sites are highly conserved within genogroups, but not across them (Cao et al., 2007; Bu et al., 2008; Tan et al., 2009). In a study by Tan et al. (2009), GI.1 (Norwalk) and GII.4 (VA387) viruses were compared, and while both strains bind the same HBGAs, types A and H, the sequences of the binding sites and the sugars involved in the interactions are unique. While both binding interfaces require P domain dimerization, the Norwalk binding site is on a single monomer, while VA387 spans the dimer. In addition to location variation, the Norwalk virus also possesses a more narrow interface, while the GII.4 has a comparatively larger and broader binding surface (Cao et al., 2007; Bu et al., 2008; Tan et al., 2008, 2009). These differences dictate how HBGAs and other components are bound by each genogroup.

## Simple Chemical Compounds that Bind Human Norovirus

Interactions of different chemical compounds with human norovirus capsids are generally investigated for two major reasons: (1) to determine if human noroviruses could be targeting other cellular receptors/co-factors (e.g., sialic acid) and (2) to identify potential antivirals that inhibit binding to cellular receptors. Heparan sulfate, citrate and sialic acid are all capable of binding human norovirus (Tamura et al., 2004; Rydell et al., 2009; Hansman et al., 2012), and may potentially play a role in norovirus pathogenesis. Glycerol, tannic acid and panels of other molecules were identified with the purpose of disrupting norovirus binding (Feng and Jiang, 2007; Zhang et al., 2012), and may have potential therapeutic application (**Table 1**). However, discussion of therapeutics and antivirals for noroviruses is beyond the scope of this review, but interested readers are referred to other reviews (Ali et al., 2016; Prasad et al., 2016; Deval et al., 2017).

**Table 1:** Norovirus binding to non-HBGA ligands.

Compound	Evidence	Reference
**Simple chemical components**		
Heparan sulfate	Molecules on host cell surface associate with GII NV	Tamura et al., 2004
Citrate	Citrate and water form a ring-like structure which mimcs the pyranoside rind of fucose	Hansman et al., 2012
Sialylated glycans	Binding of GI.3, GII.3, GII.4, to mono-, di- and tri-sialylated gangliosides with similar affinity as HBGAs	Rydell et al., 2009; Han et al., 2014; Wegener et al., 2017
Glycerol	Inhibits binding of GII.21 OIF to HBGAs	Liu et al., 2015
Tannic acid	Inhibits NV P protein binding to both A and B HBGA antigens in saliva	Zhang et al., 2012
Other screened molecules	Many molecules have been screened to determine those which disrupt NV HBGA binding	Feng and Jiang, 2007
**Complex human biological components**		
Norwalk virus attachment protein	105-kilodalton cellular binding protein	Tamura et al., 2000
Histone H1	Inhibit HBGA binding by occluding the site on NV	Tamura et al., 2003
Ileal samples	VLP binding is dependent on cell differentiation but not HBGAs	Murakami et al., 2013
Breast milk glycans	Different neoglycoconjugates bind to different norovirus strains inhibiting HBGA binding	Jiang et al., 2004; Shang et al., 2013; Weichert et al., 2016
**Food ligands**		
Lettuce/Leafy greens	Binding utilizes many different carbohydrates depending on leaf age: GalNAc, GlcNAc, sialic acid (old leaves); α-D-Gal, α-D-Man/α-D-Glc, α-L-Fuc (old and young)	Esseili et al., 2012
Oysters	Type A- and O-like HBGAs present on oyster, mussel and clam gastrointestinal cells	Tian et al., 2006, 2007
**HBGA-like moieties**		
Bacteria	*Enterobacter cloacae*, in suspension and in the BJAB cell culture system	Miura et al., 2013; Jones et al., 2014

Heparan sulfate is a cell surface proteoglycan that has been analyzed to determine if it is capable of binding human norovirus VLPs. Binding of human norovirus VLPs to intestinal 407 cells in the presence of heparin or after a heparin pretreatment to the cells was examined. Although not found in cellular membranes, heparin is structurally homologous to heparan sulfate. Investigators noticed a decrease in VLP binding in the presence of heparin when GII genogroup VLPs were used. This binding reduction was not observed in cells that were pretreated with heparin, suggesting it acts on the VLPs, not on the target cells. To further suggest heparan sulfate was responsible for VLP binding to cells observed, different lyases were introduced based on their target compounds. The ones that reduced norovirus GII VLP binding were heparan sulfate-specific: heparinase I and heparinase III. To assess whether heparin sulfate binds in the same HBGA binding pocket, differentiated Caco-2 cells, which possess H-type HBGAs and heparan sulfate on their surface, were treated with heparinase I. This reduced binding of GII VLPs to the cells by about 50%, however, it did not completely abolish it, as the VLPs were still capable of binding HBGAs on the differentiated cells. These results were conserved across different cell lines from different species and tissues, with similar results (Tamura et al., 2004). Specifically, a group of molecules called monoglycosylceramides (also called cerebrosides) have been identified to bind human norovirus GII.4 Dijon VLPs using chromatogram binding assay, fluorescence microscopy, and quartz crystal microbalance with dissipation monitoring (Bally et al., 2012). These molecules consist of a fatty acid portion, a sphingosine, and a monosaccharide. Presumably binding of VLPs would be occurring with the monosaccharide portion of the monoglycosylceramide. Binding to monoglycosylceramides was investigated because they are present in fairly high abundance in the epithelial cells of the small intestine. Interestingly, Bally et al. (2012) found some evidence suggesting that VLP binding interactions were multivalent, and required a certain degree of optimal monoglycosylceramide spacing and lipid mobility to generate a strong enough multivalent interaction for binding to occur. However, it should be noted that these observations were performed using a model lipid bilayer membrane, which does not exactly mimic the complexity of human cell membranes (Bally et al., 2012).

Citrate is commonly found in fruit and fruit products. These food items have been the subject of numerous studies investigating their health benefits, with a recent study showing citrate’s ability to reduce or inhibit the capsid’s ability to bind HBGAs (Koromyslova et al., 2015). The citrate-capsid interaction was found to structurally mimic that of the α1,2 linked fucose of HBGAs to GII.10 VLPs with comparable binding affinity as determined by X-ray crystallography and saturation transfer difference nuclear magnetic resonance, respectively (Hansman et al., 2012). More specifically, the crystal structure showed the GII.10 P domain protein interacting with the citrate molecule through seven hydrogen bonding interactions. The binding interactions with citrate were identical to those between norovirus and a terminal HBGA fucose, specifically the pyranoside ring. Competition for the same binding site was further confirmed by titrating citrate into samples binding to H type 2 antigens, as H type 2 binding signal diminished in a dose-dependent manner with increasing citrate concentration (Hansman et al., 2012). Additionally, some microscopy evidence suggested that citrate alters VLP morphology, with increased VLP diameters and ring-like structures (Koromyslova et al., 2015). Rather than inhibiting binding, these structural changes appear to make the P domain HBGA binding pocket more accessible, allowing for other potentially less specific or previously sterically inhibited molecules to bind the VLPs.

Sialic acid is a common ligand for other members of the *Caliciviridae* family [reviewed in (Newman and Leon, 2015)]. Since it is possible for different HBGAs to be sialylated, Rydell et al. (2009) investigated whether or not norovirus VLPs could bind to these altered residues. They examined sialyl Lewis x, sialyl diLewis x, and sialylated type 2 chain conjugates. They found, as with HBGAs, there is strain variation in the ability to bind the different sialylated carbohydrates, with only the GII strain VLPs showing binding (GII.3 Chron1 and GII.4 Dijon, but not GI.1 Norwalk). The sialylated carbohydrates also inhibited HBGA binding in an enzyme linked immunosorbent assay (ELISA)-based assay (Rydell et al., 2009). Additionally, evidence has been reported that human noroviruses are capable of binding gangliosides using VLPs and P protein preparations in various forms. Han et al. (2014) demonstrate that GII.4 and GI.3 human norovirus strains (VA387 and VA115, respectively) are capable of binding the oligosaccharide portions of multiple gangliosides with binding affinities comparable to HBGAs in some cases using electrospray ionization mass spectrometry. Additional evidence of human norovirus binding to these gangliosides was confirmed using enzyme-linked immunosorbent assay (ELISA) (Han et al., 2014). In another study, Wegener et al. (2017) directly compared and characterized binding of the P domain of human norovirus GII.4 MI001 to the carbohydrate portions of ganglioside GM3 (3′-sialyllactose) and an HBGA (B antigen), in addition to other carbohydrates. Interestingly, epitope mapping of 3′-sialyllactose revealed interactions between the P domain and all its carbohydrate subunits, including the sialic acid residue, despite the fact that no observation of binding to the individual monosaccharide units was observed. This was not the case for the fucosylated carbohydrates, as binding was observed for fucose methyl glycoside. The affinity of 3′-sialyllactose was found to be lower than that of the B antigen, but comparable to fucose (Wegener et al., 2017).

While the majority of work for non-HBGA ligands is done on more common genotypes, some work has been performed on emerging virus genotypes; in this case GII.21. This strain has a unique binding profile, as it does not possess the conventional conserved GII HBGA binding interface and only binds Lewis a antigen. Liu et al. (2015) investigated the structure of the P domain, observed a 90 degree flip in the P dimer conformation, and characterized a unique binding interface conserved among other GII strains. While conducting this study, glycerol was found to bind the same HBGA binding pocket of this norovirus through eight direct hydrogen bonds in a manner similar to β-galactose, a common component of HBGAs (Liu et al., 2015). Further, glycerol was found to outcompete binding of monomeric Lewis a antigen but did not inhibit binding by the corresponding multivalent complex of Lewis a, thus its utilization as a therapeutic component would require troubleshooting. As mentioned above, competition experiments are complicated by the fact that many molecules can bind the capsid in a multivalent manner.

Many molecules have been screened with the purpose of disrupting norovirus binding to HBGAs. One study specifically targeted 50 Chinese medicinal herbs commonly used to fight gastrointestinal disease. A saliva-based blocking assay using norovirus GII.4 VA387 P dimer and P particles highlighted two natural remedies effective at limiting norovirus binding, Chinese Gall and pomegranate. The compound common to these therapies is tannic acid. Pure tannic acid was obtained and compared against different forms of hydrolysable tannins to identify possible inhibitors. Of those tested, only the highly purified tannic acid inhibited binding to saliva containing both A and B type HBGAs. Although the exact mechanism remains unknown, the inhibitory effects could potentially be due to binding within the same pocket, a potential occlusion effect or potentially a conformational change (Zhang et al., 2012).

Similar to the study using Chinese herbs, Feng and Jiang (2007) screened a panel of 5,000 compounds for the ability to inhibit GII.4 VA387 VLP binding to HBGAs in saliva using an immunoassay. The screen identified 153 potential inhibitors preventing binding to HBGA A type antigen, of which 14 demonstrated strong inhibition. These compounds were checked for inhibition against other HBGAs (types B and H) and other norovirus VLPs (Norwalk, VA207 and MOH). These 14 compounds were also checked for cytotoxicity to potential host cells to evaluate their potential as therapeutics and little to no cytotoxicity was observed. In general, the different compounds’ efficacy was strain- and HBGA-specific. To adopt one of these compounds for future antiviral therapies, a more broadly effective compound or mixture would likely need to be identified (Feng and Jiang, 2007). In another chemical screening study, Rademacher et al. (2011) screened a library of 430 molecules to identify potential therapeutic compounds that bind the fucose binding site of the norovirus capsid. Multiple nuclear magnetic resonance-based techniques were serially used to screen the molecules using GII.4 VLPs, and the results used to generate and characterize inhibitory polymers. One of these contained a molecule identified in the screening process (compound 160, present in the P2 polymer), and was capable of inhibiting HBGA binding of VLPs when tested using competitive surface plasmon resonance (Rademacher et al., 2011).

## Complex Human Biological Components that Bind Noroviruses

Similar to some of the simple chemical component studies, naturally occurring human components were examined to determine if anything already present within the host is capable of inhibiting or promoting norovirus binding (**Table 1**). Both studies finding potential inhibitors to norovirus binding as well as potential attachment factors in infection have been identified in these studies.

Two of the components found were the 105-kDa Norwalk virus attachment protein and a 35-kDa histone, H1 (Tamura et al., 2000, 2003). Both of these proteins were identified using virus overlays with different norovirus VLPs. Specifically, whole cellular lysates from 293T cells (human embryonic kidney) were separated on SDS-PAGE gels and transferred to nitrocellulose membranes. These membranes were blocked and a virus overlay was completed using norovirus VLPs. First, a GII.6 Ueno virus appeared to have a strong binding interaction with a 105-kDa protein. This protein, termed Norwalk virus attachment protein, was observed in six other cell culture lines and was indispensable for the binding of other human norovirus VLPs–including the GI strains Seto and Funabashi. In general, Caco-2 cells were used to determine VLP internalization. Certain modifications to host cell surfaces suggested the interaction was protein–protein (Tamura et al., 2000). In a subsequent study, a 35-kDa protein was identified which binds to human norovirus, and was later identified as histone H1. Histone H1 had an inhibitory effect on HBGA binding for human norovirus strains from both genogroups I and II in a dose dependent manner. In addition to binding norovirus VLPs, histone H1 was found to interact with host cells, blocking potential human norovirus receptors (Tamura et al., 2003). In addition to proteins, complex glycoproteins present in human cells also have been identified to bind human noroviruses.

Human breast milk contributes to infant immunity, and some studies have suggested it plays a role in blocking the interaction of human noroviruses with HBGA receptors within the infant small intestine (Marionneau et al., 2001; Jiang et al., 2004; Ruvoën-Clouet et al., 2006; Hansman et al., 2012; Shang et al., 2013; Weichert et al., 2016). Human milk is rich in oligosaccharides found as glycoproteins, glycolipids, and free oligosaccharides, and studies have investigated the specific oligosaccharides responsible for norovirus binding inhibition. Milk from secretor and non-secretor women was tested for blocking ability to different norovirus strains. Within the milk, there were no A or B antigens, but there were secretor and Lewis antigens. Different milk compositions (secretors vs. non-secretors) were able to inhibit different virus strains, based on the binding profile of the virus (Jiang et al., 2004). Further examination determined different neoglycoconjugates may act as decoys to bind different norovirus capsids and disrupt virus binding to HBGAs. For two common norovirus strains, GI.1 Norwalk and GII.4 VA387, different breast milk inhibitors were identified using surface plasmon resonance of glycan microarrays. For GII.4 VA387, the capsid interacted with two neoglycoproteins, lacto-*N*-fucopentaose III (Lewis x-pentasaccharide) conjugated to human serum albumin and 2′-fucosyllactose conjugated to bovine serum albumin, while GI.1 Norwalk binding occurred for multiple neoglycoprotein glycans, specifically lacto-*N*-fucopentaose I (H type 1 pentasaccharide), lacto-*N*-neodifucohexaose I (Lewis x hexaose), 2′-fucosyllactose, lactodifucotetraose (difucosyl lactose), and difucosyllacto-*N*-hexaose glycine derivatives (Shang et al., 2013). Structural data has recently been reported providing a structural basis for inhibition of HBGA binding in GII.10 capsids for two identified human milk oligosaccharides, 2′-fucosyllactose and 3′-fucosyllactose, both of which could effectively inhibit HBGAs in porcine gastric mucin and A and B type saliva from binding. As mentioned for other compounds above, these oligosaccharides interacted with the HBGA binding pocket, with the fucose moieties of the oligosaccharides positioned in the same manner as the fucose moieties on HBGAs (Weichert et al., 2016).

## HBGA-Like Moieties On Foods and Microflora

Although primarily transmitted through food handlers and complex prepared foods, foodborne human norovirus outbreaks have occurred with specific food products as well, primarily produce and shellfish (Batz et al., 2012). The higher incidence rates of these specific foods raised questions as to whether binding is occurring between different cell types in the foods and norovirus and if such interactions are specific. Leafy greens are more frequently implicated in norovirus outbreaks than other simple foods, likely because they are often picked/processed by hand, potentially irrigated with contaminated water, and are commonly lightly cooked or eaten raw. Researchers examined leafy greens for the presence of HBGAs and their related carbohydrates. Lectins were used to determine the specific sugar residues involved, specifically those with A, B, H type 2, and Lewis y activity. Other sugars were tested due to their presence in HBGAs, or as controls looking at the specificity of the ligand binding. Esseili et al. (2012) discovered that human norovirus VLPs attach directly to lettuce leaves using immunofluorescence and ELISA, usually along veins, stomata or tears. Binding was specific to cell wall carbohydrates associated with sugars found in HBGAs, instead of simpler forms of carbohydrates (i.e., mannose). Exposing the cell wall carbohydrates to lectins targeting multiple carbohydrates (α-D-Gal, α-D-Man/α-D-Glc, and α-L-Fuc) resulted in reduced GII.4 VLP binding. Interestingly, a difference was observed in the carbohydrate composition of old leaves and young leaves; as old leaves exhibited a higher degree of binding to norovirus GII.4 VLPs, and reduced binding was only observed with older leaves upon exposure to lectins targeting GalNAc, GlcNAc, and sialic acid. These specific binding interactions likely aid the viral persistence and attachment on leaf surfaces (Esseili et al., 2012) (**Table 1**).

Molluscan shellfish are other simple food items commonly implicated in norovirus outbreaks. These shellfish (i.e., oysters, mussels, and clams) are filter feeders, capable of concentrating viral particles from contaminated water in their guts. While it is possible viral concentration is due solely to filter feeding, accumulation of norovirus could be due to more specific interactions that would have consequences on the practice of depuration. An initial study found HBGA-like residues in oyster gastrointestinal tracts. Utilizing anti-HBGA antibodies in an ELISA-based assay, it was determined that type A-like HBGAs are found in these tissue samples. This finding was confirmed using immunofluorescent histochemical staining (Tian et al., 2006). Another study by Tian et al. (2007) expanded its scope to clams, mussels and oysters. As previously reported, oysters possessed type A-like moieties, but it was found they also possess type O-like compounds. This type A-like binding was found on the mussels and clams tested, however, only some of those shellfish tested possessed the type O-like antigen as well. This study found these bivalves are capable of binding both genogroups I and II, as observed by immunofluorescent microscopy (Tian et al., 2007). Further investigation into the interactions of different genogroup VLPs (GI.1 Norwalk and GII.4 Houston) with oysters suggested that GII.4 VLPs were additionally capable of binding sialic acid-like structures present in oysters using lectins and neuraminidase to reduce VLP binding (Maalouf et al., 2010). These differences in carbohydrate ligand specificities that were observed have also been hypothesized to contribute to strain- and tissue-dependent differences in norovirus bioaccumulation observed between GI.1, GII.3, and GII.4 genotypes (Maalouf et al., 2011).

Like molluscan shellfish, the presence of HBGA/HBGA-like moieties on bacteria had long been demonstrated (Springer et al., 1961). In light of the recent discoveries with molluscan shellfish, research then focused on potential norovirus binding to bacteria found in the human gut, the suspected site of norovirus infection. Miura et al. (2013) demonstrated that the gut bacteria *Enterobacter cloacae* bound human norovirus in suspension, and that this interaction was due to HBGA-like residues found on the bacteria—specifically type H-like moieties. This finding was confirmed via ELISA and transmission electron microscopy, showing noroviruses from both genogroups I and II were capable of these interactions (Miura et al., 2013). This was also observed with lactic acid bacteria using P particles (Rubio-del-Campo et al., 2014). Furthermore, a putative cell culture system suggested that synthetic HBGAs or heat-killed *E. cloacae* were needed to elicit productive infection in a human B cell line and B cells co-cultured with intestinal epithelial cells (Jones et al., 2014). Whether these findings are unique to *E. cloacae* or a more widespread phenomenon involving other bacteria remains to be seen. However, another study demonstrated a reasonable degree of binding of two GII.4 (Sydney and New Orleans) and one GI (GI.6) strain to a panel of ten enteric bacteria, demonstrating some degree of binding to all the bacteria, albeit at different levels. Some of the bacteria included were isolated from human stool samples and identified by sequencing (Almand et al., 2017). Additionally, Almand et al. (2017) demonstrated that bacterial binding to human norovirus (and presumably expression of HBGA-like moieties) was significantly affected by culture conditions, as richer supplemented media reduced binding while use of minimal media produced the highest human norovirus binding. Interestingly, these bacteria-norovirus interactions may have future viral concentration and detection value, as bacteria were found to remove viruses from solution (concentrate) at fairly high efficiencies, sometimes above 95% for some bacteria (Almand et al., 2017). This concept was first introduced for noroviruses by Amarasiri et al. (2016) in the context of enhancing membrane filtration to remove noroviruses from water, with positive results in varying degrees based on bacterial strain. Norovirus-bacteria interactions have also been suggested to have consequences for control of foodborne norovirus transmission. Li et al. (2015) found that norovirus binding to two *Escherichia coli* strains with HBGA-like molecule activity significantly reduced the efficacy heat treatment (90°C, 2 min) compared to an *Escherichia coli* strain without HBGA-like molecule activity.

## Conclusion

There is a vast amount of literature on HBGAs and related carbohydrates and their interactions with human noroviruses. Less studied, however, are other molecules and cell-types that directly bind norovirus. Reviewing these alternative binding partners is important given the evidence that additional unknown co-factors/attachment factors may be involved in the norovirus infection process, as well as the potential therapeutic importance of alternative ligands that may occlude HBGA binding and inhibit infection. Certainly, there is more research to be done regarding these alternative ligands, and future research should be conducted to further elucidate their structures and nature of interaction with human norovirus. Much like the HBGA-like moieties on gut bacteria, further research into these additional ligands may have downstream physiological or therapeutic importance. Additionally, these different ligands prove useful in the study and concentration/detection of human norovirus, as many of them are cheaper and more readily available than synthetic HBGA carbohydrates. In summary, multiple additional non-HBGA ligands for human noroviruses have been discovered, and further research into them may better inform future detection, control, and study of human noroviruses.

## Author Contributions

EA conceived, wrote the initial draft, and edited the manuscript. MM revised, edited, and submitted the manuscript. L-AJ conceived, revised, edited, supervised, and provided funding for the manuscript.

## Conflict of Interest Statement

The authors declare that the research was conducted in the absence of any commercial or financial relationships that could be construed as a potential conflict of interest.
